# New Insights into the Migration Characteristics of Polymer Systems in Porous Media

**DOI:** 10.3390/polym18050568

**Published:** 2026-02-26

**Authors:** Lijuan Zhang, Shutong Li, Xiqun Tan, Jirui Zou, Renbao Zhao, Yuan Yuan, Xiang’an Yue

**Affiliations:** 1 College of Petroleum Engineering, China University of Petroleum (Beijing), Beijing 102249, China; 2 Exploration and Development Research Institute of Changqing Oilfield Company, Xi’an 710021, China; 3 College of Energy Innovation, China University of Petroleum (Beijing), Beijing 102249, China; 4 National Key Laboratory of Heavy Oil, China University of Petroleum (Beijing), Beijing 102249, China

**Keywords:** EOR, polymer flooding, migration pattern, hydrodynamic radius, viscosity, throat–polymer ratio

## Abstract

Knowledge of the migration characteristics of polymer systems in pore throats is essential for the effective application of polymers as a profile-control oil-displacement agent for enhanced oil recovery. In this study, the effect of concentration on the viscosity and hydrodynamic radius of polymer systems was investigated using a rheometer and a dynamic light scattering instrument. Furthermore, pore-throat models, homogeneous cores, and multi-measuring-point sand-packed models were constructed to investigate pore-scale migration patterns and the effect of the throat–polymer ratio (defined as the ratio of throat size to polymer hydrodynamic radius) on the migration properties of polymers in porous media. The results showed that the transport of polymer systems in porous media is primarily related to the throat–polymer ratio. When this ratio is sufficiently small (i.e., no more than 18.94), the migration pattern of the polymer systems in the pore-throat model does not exhibit the characteristics of polymer solution flow, but rather, of discontinuous-dispersion retention, plugging-breakthrough migration, and stable-plugging retention. Upon increasing the injection rate, the polymer systems also exhibit the migration characteristics of discontinuous dispersion at a larger throat–polymer ratio. Moreover, polymer system migration resistance and improved sweep efficiency in porous media are influenced by not only the viscosity of polymer systems, but also the throat–polymer ratio. The smaller the throat–polymer ratio, the stronger the retention and plugging ability of the polymer systems. The outcomes of this study are significant for the design of polymer flooding operations in oilfields.

## 1. Introduction

Polymer flooding technology is an important method for enhancing oil recovery, and its application in China’s Daqing, Shengli, and Bohai oilfields has achieved good oil stabilization and water control [1,2,3,4]. It is generally believed that the main mechanism of polymer flooding involves using a polymer to improve the viscosity of the displacement liquid, improve the mobility ratio between the displacement and displaced phases to inhibit viscous fingering, and improve the sweep efficiency, with the aim of enhancing oil recovery [5,6,7,8,9,10]. In more recent studies, researchers have realized that the viscoelasticity of polymer systems could improve oil displacement efficiency and reduce residual oil saturation [11,12,13,14,15,16].

Based on the main mechanism of polymer flooding, the viscosity of a polymer solution has become the primary technical index for evaluating polymer performance. In addition, researchers have determined that the higher the viscosity of a polymer solution, the better the oil displacement effect of the polymer. However, experimental results indicate that high-viscosity polymer systems are likely to exhibit poor transport in porous media [17,18]. In particular, polymer systems with excessively high viscosity may lead to injection problems due to the formation of oversized polymer clusters [19,20,21,22,23]. It is worth noting that the flow of a polymer system in a reservoir is dependent on the inlet–outlet end-face effect when the fluid flows through numerous pore throats, which is considerably different from the standard flow field in the rotating cylinder of a rheometer. In addition, experimental data show that the viscosity of a polymer system flowing in the micro-scale pores of a reservoir is different from that measured by a rheometer in a laboratory [24,25,26,27,28,29,30,31,32,33]. For example, Yue found that polymer flow in microtubes has a drag-reducing effect, which increases with decreasing tube diameter and shear velocity. The flow rate of polymers in 10.1 μm microtubes is 4.19 times higher than that in conventional large-scale environments [25]. Wei found that the effective viscosity of a polymer in a core was 59.37% of the viscosity measured by a viscometer [26], and Sagyn Omirbekov found that the viscosity of guar gum measured in porous media was higher than that measured in a rheometer [32]. In addition, a study by Dauben revealed that high-molecular-weight poly(ethylene oxide) solutions exhibit rheological behavior in glass-bead-packed artificial porous media, which is the complete opposite of what was observed in simple flow fields. While these solutions behave as typical pseudoplastic (shear-thinning) fluids in a rotational rheometer, they display dilatant (shear-thickening) flow behavior in porous media, characterized by an anomalous increase in flow resistance with rising flow rate. This phenomenon is governed by flow velocity, pore-throat size, polymer molecular weight, and concentration; moreover, such elevated flow resistance cannot be predicted from viscosity measurements alone [34]. There are two possible reasons for the difference between bulk viscosity and viscosity in porous media: (i) adsorption and mechanical retention of the polymer alter the pore geometry of the porous medium [35]; (ii) non-negligible extension-thickening behavior triggers elastic turbulence, which may arise from a positive feedback loop between polymer chain stretching and the flow field [36], or from the impact of high-stress birefringent strands on the flow pattern [37]. Therefore, viscosity parameters measured by a rheometer cannot accurately describe the dynamic behavior of porous media’s migration in reservoir pores.

It is particularly noteworthy that the size of the polymer molecular clusters used for oil displacement is similar to that of the micron-scale pore throats of a reservoir, and it is unknown whether a polymer system can act as a continuum when it migrates in a micron-scale pore throat. When the size of a molecular cluster approaches that of pore throats, the polymer solution may no longer behave as a continuum. Campo-Deaño et al. observed that when a polyacrylamide solution flows through porous media with silica surfaces, adsorption-induced nanogelation occurs, leading to plugging when the interparticle spacing is sufficiently small. This results in a much steeper increase in the pressure drop curve than that predicted by viscoelastic effects alone [38]. If a polymer system no longer presents the flow characteristics of the polymer solution when flowing in the micropores of a reservoir, what other factors will affect the sweep efficiency of polymer flooding in addition to the system viscosity? In summary, it is debatable whether we should use viscosity as the only performance index to evaluate the oil displacement effect of polymer systems, and to improve the mobility ratio, which is the primary mechanism by which the polymer system expands the swept volume. In this work, the effect of polymer concentration on the viscosity and hydrodynamic radius of a polymer system was first investigated. Then, the effects of throat–polymer ratio and injection rate on the migration pattern were systematically investigated through a series of pore-throat model migration experiments. Next, polymer system flow experiments in porous media were carried out to study the effect of throat–polymer ratio on the flow of the polymer system in a core and a multi-measuring-point sand-packed model. Finally, the mechanism of polymer system migration in reservoir porous media is expounded. The outcomes of this study would be helpful for designing polymer flooding operations in oil reservoirs.

## 2. Experimental Section

### 2.1. Materials

**Figure 1:**
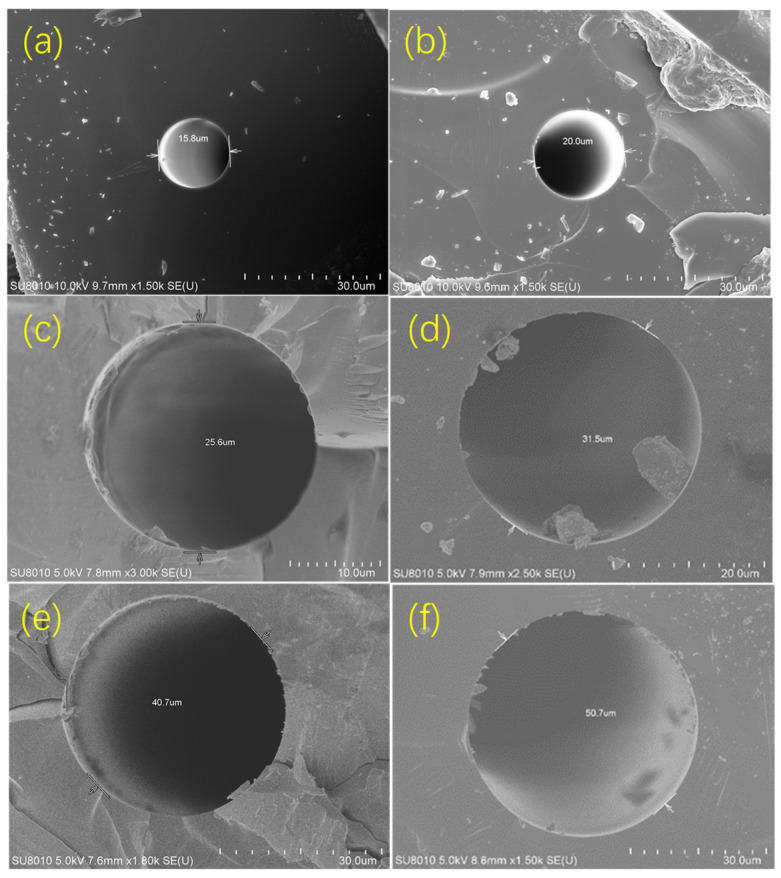
Electron microscope scans of microtubules ((**a**) 15 μm; (**b**) 20 μm; (**c**) 25 μm; (**d**) 30 μm; (**e**) 40 μm; (**f**) 50 μm).

HPAM with a molecular weight of 27.33 million and a degree of hydrolysis of 26.8% was provided by Beijing Hengju Chemical Group Co., Ltd., Beijing, China and xanthan gum with a molecular weight of 1 million was provided by Henan Spring Xiangrui Chemical Co., Ltd., Xinxiang, China. Deionized water was used as the experimental water. Silica microtubes (Polymicro Technologies company, Phoenix, AZ, USA) with nominal inner diameters ranging from 15 μm to 50 μm were used in the experiments to fabricate the pore-throat model. The end faces of the microtubes were examined using a scanning electron microscope (Hitachi, SU8010, Tokyo, Japan); the obtained images (Figure 1) indicate that the microtubules used in the experiment were standard circular tubes, and their actual inner diameters were consistent with the nominal values.

### 2.2. Methods

#### 2.2.1. Viscosity Measurement Method

The viscosities of HPAM and xanthan gum were measured using a rheometer (HAAKE RS6000, HAAKE, Karlsruhe, Germany) at a shear rate of 7.34 s^–1^ and a temperature of 26 °C.

#### 2.2.2. Measurement of the Hydrodynamic Radius of the Polymer System

The hydrodynamic radius of the polymer system was measured using a dynamic light scattering instrument (ALV CGS-3, ALV, Langen, Germany) at a temperature of 26 °C.

#### 2.2.3. Morphology of HPAM System in Water

The HPAM system was completely frozen in liquid nitrogen (−180 °C), quickly transferred to a vacuum freeze dryer (YTLG-10A, YTLG, Shanghai, China), and vacuumed for 24 h. The microscopic morphology of the HPAM system was observed using an environmental scanning electron microscope (FEI Quanta 200, FEI, Eindhoven, The Netherlands).

#### 2.2.4. Experiment on HPAM Migration in the Pore-Throat Model

**Figure 2:**
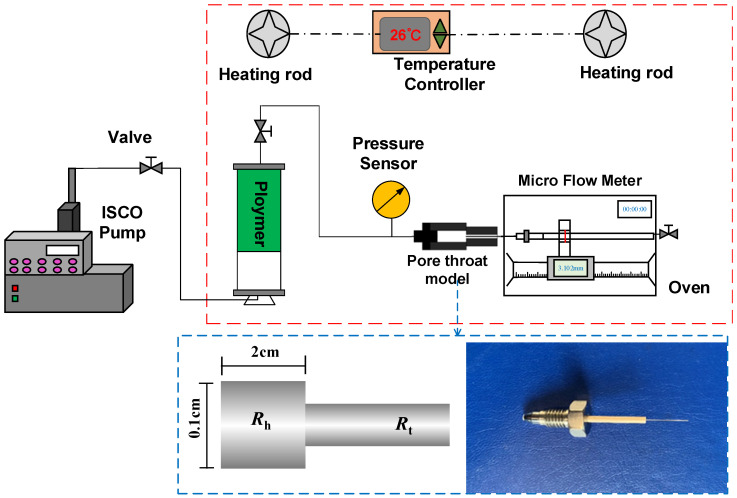
Schematic diagram of the experiment on HPAM migration in the pore-throat model.

In this study, the migration characteristics of HPAM in the pore-throat model were investigated based on the injection pressure and flow rate. The experiments were performed according to the experimental setup shown in Figure 2, with the pore length and pore radius in the pore-throat model measuring 2 cm and 0.1 cm, respectively. The key parameters in the experiments are shown in Table 1, including the throat radius, throat length, hydrodynamic radius of HPAM, and injection rate and throat–polymer ratio (*R_tp_*) in each experiment. The throat–polymer ratio is defined as the ratio of the throat radius to the hydrodynamic radius of HPAM, which characterizes the relative size of the HPAM cluster and throat size.

The experimental steps were as follows: (a) An HPAM solution of 1000 mg/L was prepared with deionized water and aged for 12 h, and 1000 mg/L HPAM was diluted to 100 mg/L and 50 mg/L for the experiments. Each prepared polymer system was filtered through a nuclear microporous filter membrane with a pore size of 5 μm to remove impurities and dust. (b) Experimental pipelines, intermediate vessels, and pore-throat models were purged with high-purity nitrogen to remove dust and impurities. (c) The HPAM solution in the intermediate vessel was injected into the pore-throat model at a constant flow rate using an ISCO pump, and the injection pressure and flow rate at the outlet end of the pore-throat model were recorded during the experiment.

#### 2.2.5. Experiment on HPAM Migration in the Core

**Figure 3:**
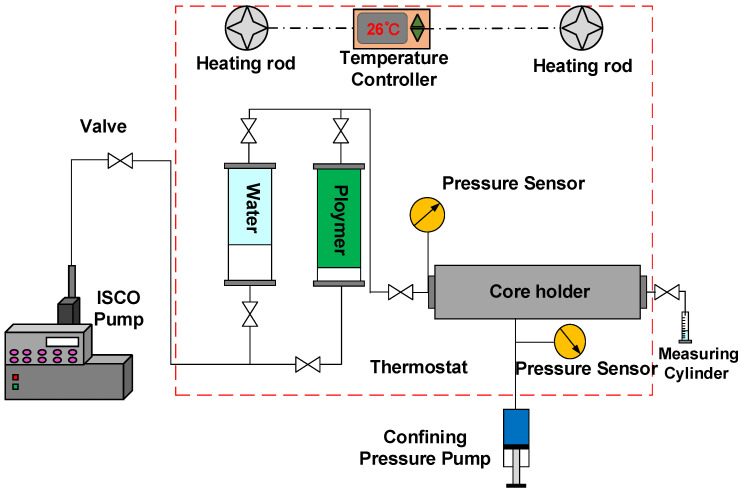
Schematic diagram of the experiment on polymer system migration in cores.

The experiment was carried out according to the diagram shown in Figure 3, with the core size set to Φ2.5 × 10 cm. Table 2 shows the parameters of the cores. The average throat radius $R_{t}$ in Table 2 was calculated as follows: [39]

(1) $$R_{t}=\sqrt{\frac{8k}{\emptyset }}$$

where $R_{t}$ is the average throat radius, μm; *k* is the permeability, μm^2^; and ∅ is the porosity.

The experimental steps were as follows: (a) The cores were dried at 80 °C for 12 h and their dry weights were measured. (b) The cores were evacuated for 12 h, then saturated with deionized water and soaked for 24 h, and the wet cores were weighed to calculate the porosity. (c) Water was injected at a flow rate of 0.8 mL/min until the injection pressure was stable, and the permeability of the cores was measured. (d) HPAM was injected at the same flow rate until the injection pressure was stable, and the resistance coefficient was calculated as follows:

(2) $$F_{R}=\frac{∆p_{p}}{∆p_{w}}$$

where $F_{R}$ is resistance coefficient; $∆p_{p}$ is the stable pressure of polymer displacement, KPa; and $∆p_{w}$ is the stable pressure of water displacement, KPa.

(e) The deionized water was injected at the same flow rate until the injection pressure was stable, and the residual resistance coefficient was calculated as follows:

(3) $$F_{RR}=\frac{∆p_{sw}}{∆p_{w}}$$

where $F_{RR}$ is the resistance coefficient; $∆p_{sw}$ is the stable pressure of subsequent water displacement, KPa; and $∆p_{w}$ is the stable pressure of water displacement, KPa.

#### 2.2.6. Experiment on HPAM and Xanthan Gum Migration in the Sand-Packed Model

**Figure 4:**
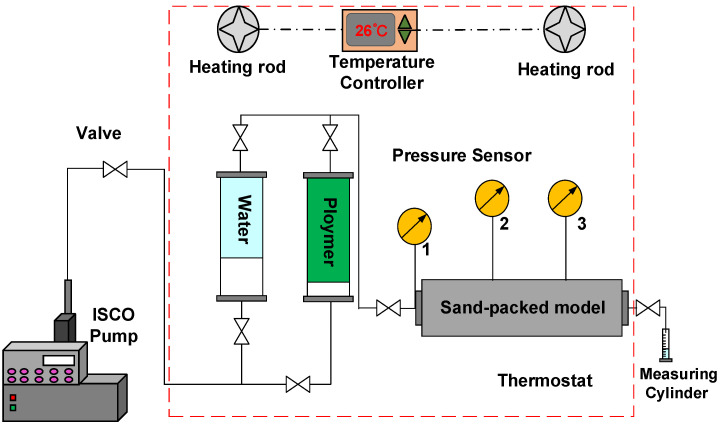
Schematic diagram of the migration experiment of polymer system in sand-packed models.

The experiment was conducted using the setup shown in Figure 4, and the parameters of the sand-packed models are listed in Table 3. The average throat radius of the sand-packed model was calculated using Equation (1). The experimental steps were as follows: (a) The dry weights of the sand-packed models filled with quartz sand were measured. (b) The sand-packed models were evacuated for 12 h, then saturated with deionized water and soaked for 24 h, and the wet sand-packed models were weighed to calculate the porosity. (c) Deionized water was injected at a flow rate of 0.5 mL/min until the injection pressure stabilized, and the permeability of the sand-packed models was measured. (d) HPAM or xanthan gum was injected at the same flow rate until the injection pressure was stable, and the resistance coefficient was calculated using Equation (2). (e) Deionized water was again injected at the same flow rate until the injection pressure stabilized, and the residual resistance coefficient was calculated using Equation (3).

## 3. Results and Discussion

### 3.1. Effect of HPAM Concentration on Hydrodynamic Radius and Viscosity

**Figure 5:**
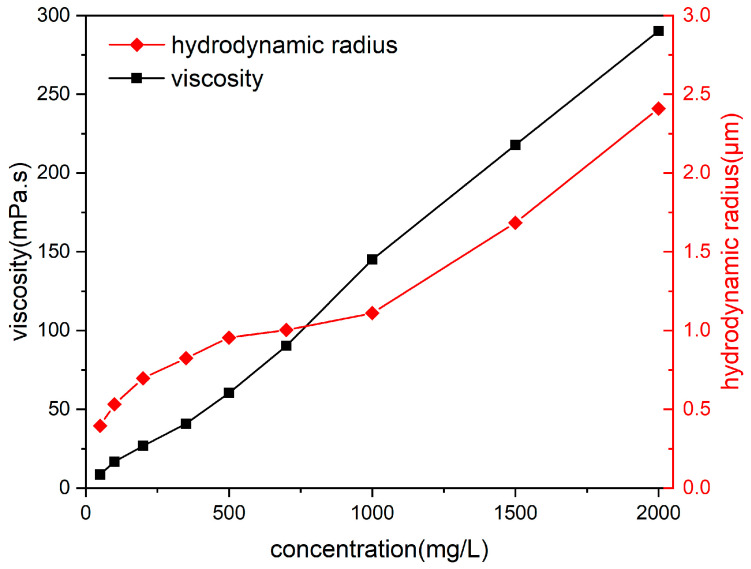
Effect of HPAM system concentration on hydrodynamic radius and viscosity.

Figure 5 shows the hydrodynamic radius and viscosity of the polymer system as a function of concentration. Figure 5 demonstrates that both the hydrodynamic radius and viscosity of the HPAM system increase with an increase in concentration. When the concentration of the HPAM system was increased from 50 mg/L to 2000 mg/L, the corresponding hydrodynamic radius increased from 0.396 μm to 2.40 μm, and the viscosity increased from 8.59 mPa·s to 290.2 mPa·s.

**Figure 6:**
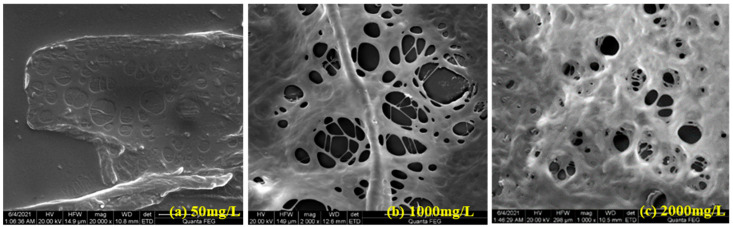
Micromorphology of polymer systems with different concentrations ((**a**) 50 mg/L; (**b**) 1000 mg/L; (**c**) 2000 mg/L).

Figure 6 shows the morphology of HPAM systems at different concentrations in water. As shown in Figure 6, at higher HPAM concentrations, the molecular chains in the aqueous solution are more prone to entanglement, leading to a denser structure and an increase in the hydrodynamic radius of the HPAM system. The friction between the corresponding liquid layers increases, and therefore, so does the viscosity of the HPAM system [40].

### 3.2. Effect of Throat–Polymer Ratio on Migration Characteristics of HPAM Systems in Pore-Throat Model

It is well known that the migration characteristics of the dispersion system in porous media differ from those of a solution. When transported in porous media, the dispersion system can temporarily or permanently block the pore space through bridging, resulting in fluctuating changes or a continuous rise in injection pressure. Some researchers have found that the relative size of the particles in the dispersed system and the pore size of the porous medium determine the transport properties of the dispersed system in the porous medium [41,42,43,44]. When the pore size is much larger than the particle size, the injection pressure of the dispersed system is smooth and shows no fluctuations. Only when the difference between pore size and particle size is small does the injection pressure of the dispersed system exhibit fluctuating variations or continuously increasing transport characteristics. This raises the question of whether a polymer with a certain hydrodynamic radius will have the same migration characteristics as the dispersion system when migrating in a sufficiently small pore.

**Figure 7:**
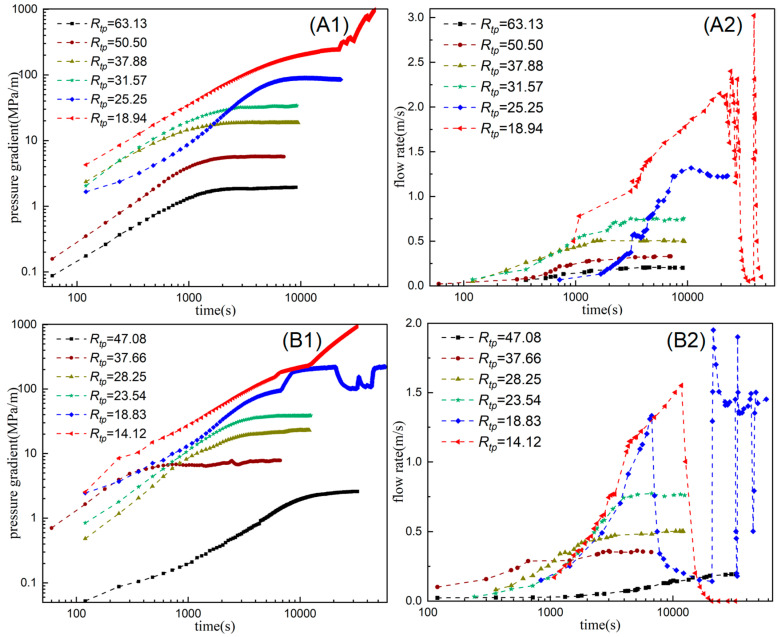
Changes in injection pressure and flow rate with injection time under different *R_tp_* values ((**A1**) The pressure gradient of the 50 mg/L polymer system varies with time; (**A2**) The flow rate of the 50 mg/L polymer system varies with time; (**B1**) The pressure gradient of the 100 mg/L polymer system varies with time; (**B2**) The flow rate of the 100 mg/L polymer system varies with time).

Experiments on migration of the HPAM system in pore-throat models were conducted using models with different throat sizes. Figure 7 shows the injection pressure and flow rate as functions of injection time for two HPAM systems with different concentrations at different throat–polymer ratios *R_tp_* (the ratio of the throat radius to the hydrodynamic radius of the polymer system). In the first part of the experiment (serial numbers 1~6), an HPAM system with a concentration of 50 mg/L was used with a hydrodynamic radius of 0.396 μm. The throat radius of the pore-throat model is 7.5 μm~25 μm, and the corresponding throat–polymer ratio (*R_tp_*) is 18.94~63.13. The experimental results are shown in Figure 7(A1,A2). When *R_tp_* is large (63.13, 50.50, 37.88, 31.57, 25.25), with an increase in injection time, the injection pressure and flow rate rise steadily to the limit and remain stable. At this time, the migration of the HPAM system in the pore-throat model shows the flow characteristics of the HPAM solution. On the other hand, when *R_tp_* is small (e.g., 18.94), as injection time increases, the injection pressure and flow rate begin to exhibit obvious fluctuations after rising to a certain value. This pronounced fluctuation is caused by the retention and blockage-breakthrough migration behavior of HPAM clusters. When the throat is blocked by HPAM clusters, the injection pressure increases and the flow velocity at the outlet drops sharply. When the pressure difference increases sufficiently, the polymer clusters break through the throat and migrate downstream, causing the pressure to drop sharply and the flow velocity at the outlet to increase sharply. The migration of HPAM systems in the pore-throat model is characterized by discontinuous-dispersion retention and blockage-breakthrough migration.

To investigate the migration characteristics of the polymer system in pore throats with a smaller throat–polymer ratio, in the second part of the experiment (serial numbers 7~12), an HPAM system with a concentration of 100 mg/L was used with a hydrodynamic radius of 0.531 μm. In this case, when the throat radius of the pore-throat model ranges from 7.5 μm to 25 μm, the corresponding throat–polymer ratio (*R_tp_*) ranges from 14.12 to 47.08. The experimental results are shown in Figure 7(B1,B2). Similarly to the results obtained in the first part of the experiment, when *R_tp_* is large (47.08, 37.66, 28.25, 23.54), the migration of the HPAM system in the pore-throat model shows the flow characteristics of the HPAM solution. When *R_tp_* is small (18.83, 14.12), the migration of the HPAM system in the pore-throat model is characterized by intermittent dispersion behavior. It is particularly noteworthy that when *R_tp_* is 14.12, the throat is stably blocked by HPAM clusters, the injection pressure continues to rise, and the flow velocity at the outlet end of the model drops to zero. The migration of the HPAM system in the pore-throat model is characterized by stable plugging of discontinuous dispersion. This may be because polymer molecules coalesce and remain at the throat when passing through it at high speed. In summary, the throat–polymer ratio (*R_tp_*) is an important parameter that affects the migration mode of polymer systems in pore throats. When the throat–polymer ratio is sufficiently small, the migration pattern of the polymer system in the pore-throat model does not exhibit the characteristics of polymer solution flow. Instead, it displays behavior consistent with intermittent retention and plugging, characterized by either breakthrough migration or stable plugging. In this study, when *R_tp_* > 18.94, the migration of the HPAM system in the pore-throat model is characterized by the flow of polymer solution. When 14.12 < *R_tp_* < 18.94, the migration of the HPAM system in the pore-throat model is characterized by the retention of plugging-breakthrough of the dispersion. When *R_tp_* < 14.12, the migration of the HPAM system in the pore-throat model is characterized by the retention of stable blockage of the dispersion.

### 3.3. Influence of Injection Rate on Migration Characteristics of HPAM Systems in Pore-Throat Model

**Figure 8:**
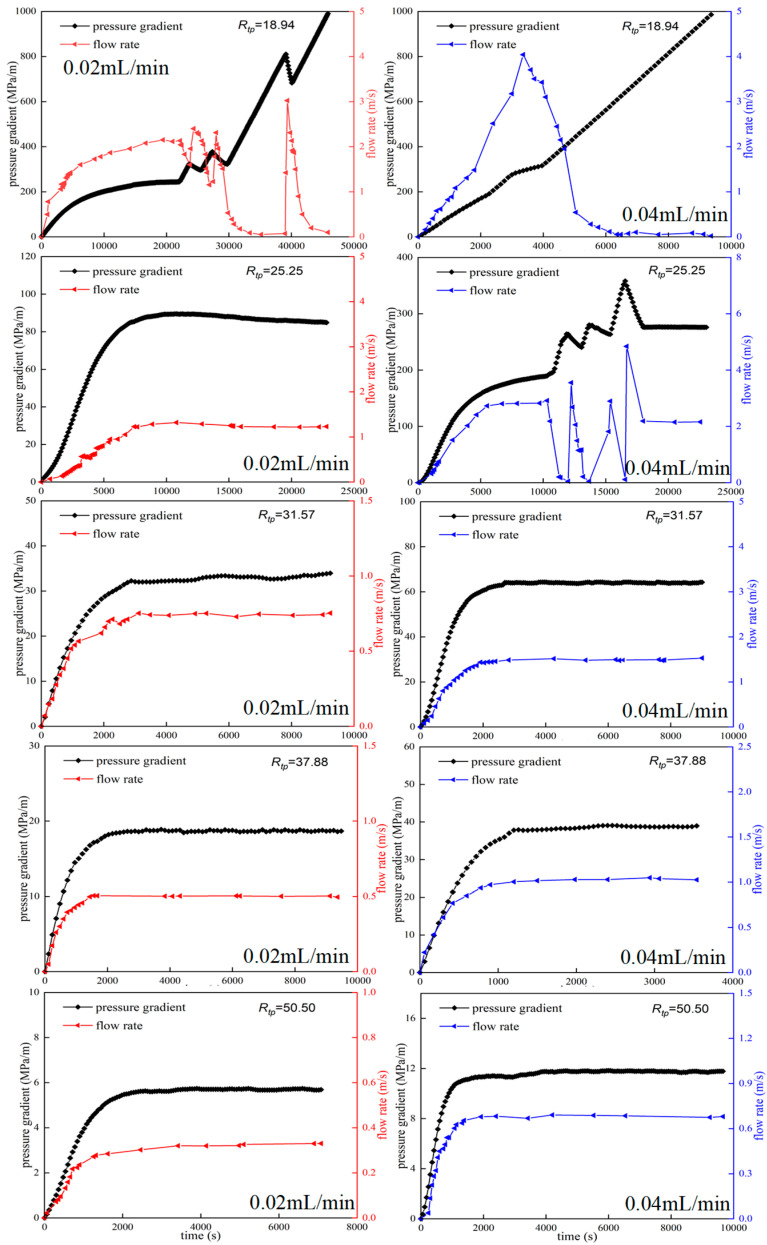
Effect of injection rate on migration characteristics of HPAM in pore-throat model (50 mg/L).

**Figure 9:**
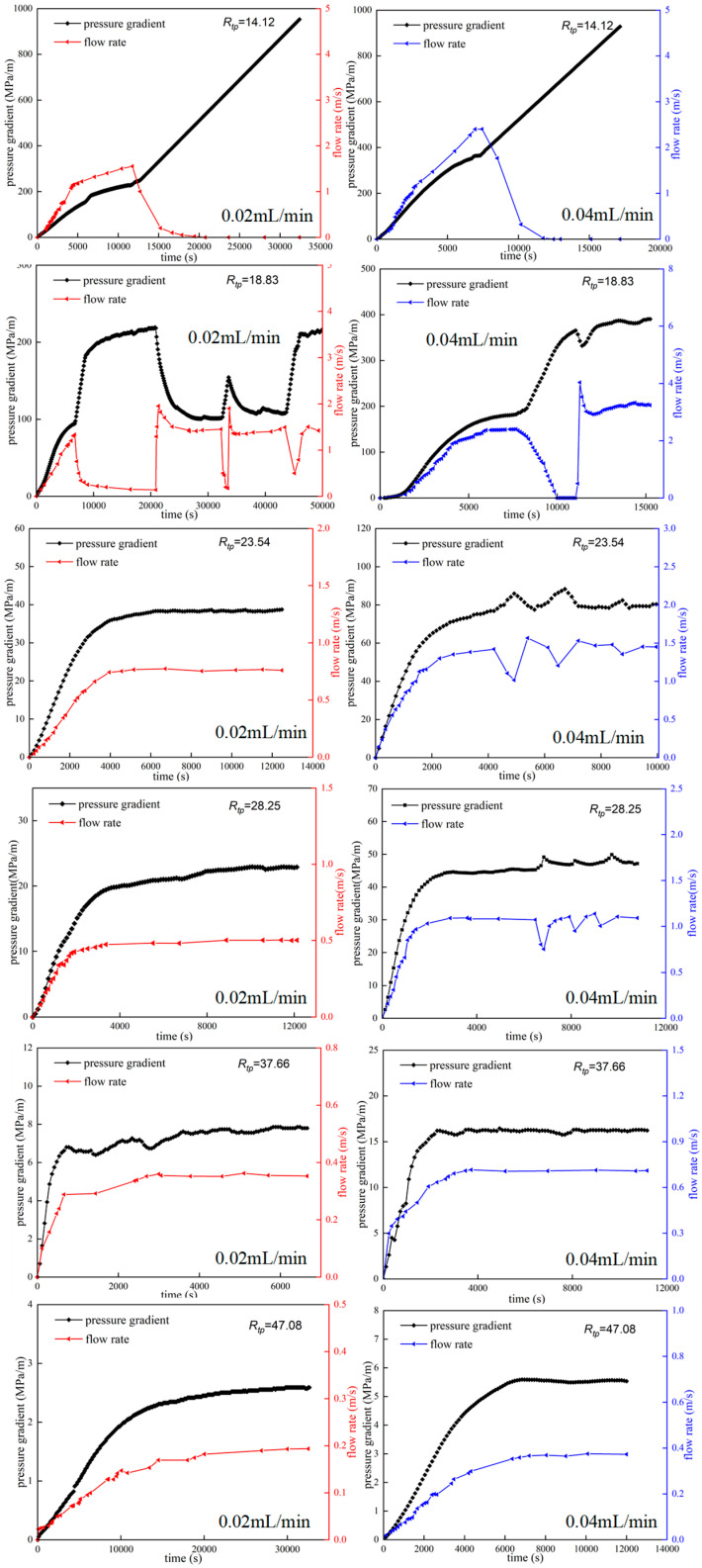
Effect of injection rate on migration characteristics of HPAM in pore-throat model (100 mg/L).

Figure 8 shows the migration characteristics of the 50 mg/L HPAM system in the pore-throat model at different injection rates. Figure 8 shows that at an injection rate of 0.02 mL/min, only when *R_tp_* is reduced to 18.94 do the injection pressure and flow rate have obvious random fluctuations. The migration of the HPAM system presents the characteristics of polymer clusters, such as dispersion, retention at pore throats, and blocking-breakthrough migration. However, at an injection rate of 0.04 mL/min, when *R_tp_* is reduced to 25.25, the injection pressure and flow rate exhibit obvious random fluctuations. When *R_tp_* drops to 18.94, the injection pressure continues to rise and the flow rate drops to zero. The migration of the HPAM system exhibits the characteristics of stable polymer-clusters blockage, such as dispersion at pore throats. Similar experimental results were obtained when HPAM at a concentration of 100 mg/L was transported in the pore-throat model (Figure 9). When the injection rate was 0.02 mL/min, the HPAM system showed the migration characteristics of the dispersed system only when the throat–polymer ratio was no higher than 18.83. When the injection rate was increased to 0.04 mL/min, the *R_tp_* corresponding to the migration characteristics of the dispersion system increased to 28.25. At high flow rates, the sharp convergence of streamlines at the pore throat entrance generates a strong extensional flow field, in which polymer molecules lack sufficient time to adjust their conformation to the flow. This causes them to deviate from the central path and collide with pore walls, leading to retention and ultimately shifting the migration pattern toward dispersed behavior. In summary, the high injection rate will cause the HPAM system to exhibit the migration characteristics of discontinuous dispersion even at larger throat–polymer ratios. It is worth noting that the critical throat–polymer ratio value is likely dependent on several factors, including the polymer type; molecular weight; chain flexibility; wettability and mineral composition of the porous medium; and salinity. We intend to further investigate the influence of these factors in future work.

### 3.4. Effect of Throat–Polymer Ratio on Migration Characteristics of Polymer Systems in Porous Media

**Figure 10:**
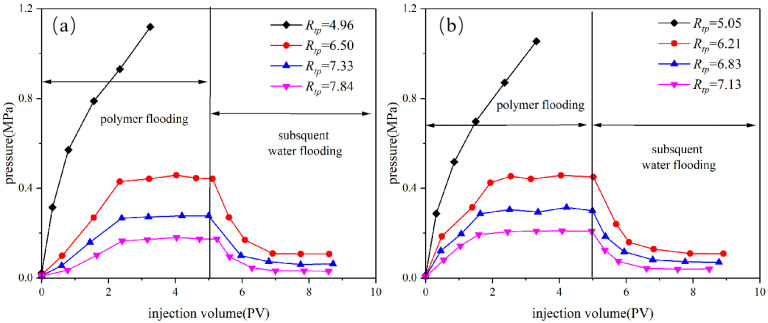
Dynamics of injection pressure of HPAM system in core ((**a**) 50 mg/L; (**b**) 100 mg/L).

Figure 10 shows the variation in injection pressure with injection volume for polymer systems at two different concentrations in cores. Table 4 lists the corresponding resistance coefficients and residual resistance coefficients. Figure 10a and Table 4 demonstrate that for the polymer system with a concentration of 50 mg/L, when the throat–polymer ratio is small (4.96), the injection pressure continues to increase as the injection volume increases, indicating that the core is blocked by the injected polymer system. The higher injection pressure was caused by the blocking of the polymer clusters at the core inlet face. When the throat–polymer ratio is increased to 6.50, the matching degree between the polymer system and the core pore throat is considerably improved. At this time, the injection pressure increases with an increase in the injection amount, and finally reaches stability. During the migration of the polymer system, many polymer clusters become trapped in the pore throats of the core, resulting in high resistance coefficients and residual resistance coefficients. However, when the throat-to-polymer ratio is too high (e.g., 7.84), the polymer clusters cannot be effectively retained in the excessively large pore throats, leading to a decrease in flow resistance and a corresponding reduction in the resistance coefficient during polymer injection. In addition, the scour resistance of the subsequent water flooding is weakened, and the residual resistance coefficient decreases. As shown in Figure 10b and Table 4, the polymer system with a concentration of 100 mg/L exhibited obvious blocking behavior when *R_tp_* was 5.05. When the throat–polymer ratio is large (6.21–7.13), the resistance coefficient and residual resistance coefficient decrease with an increase in the throat–polymer ratio. This shows that the injection ability of the polymer system increases with an increase in *R_tp_*, but the anti-scour ability decreases. Xie reached a similar conclusion, identifying a critical ratio of 4 to 7 between the polymer’s hydrodynamic radius and the median pore radius of the core; exceeding this ratio leads to polymer plugging [23]. In summary, the throat–polymer ratio plays a critical role in determining the migration behavior of polymer systems in porous media. As the throat–polymer ratio decreases, the retention capacity and plugging capacity of the polymer system increase. Only when there is a good match between the hydrodynamic radius of the polymer system and the size of the pore throat, can the polymer system effectively migrate to the deepest part of the reservoir and effectively block high-permeability channels. In this study, it was found that when *R_tp_* is no more than 5.05, the polymer system and the core pore-throat size are considerably mismatched.

**Figure 11:**
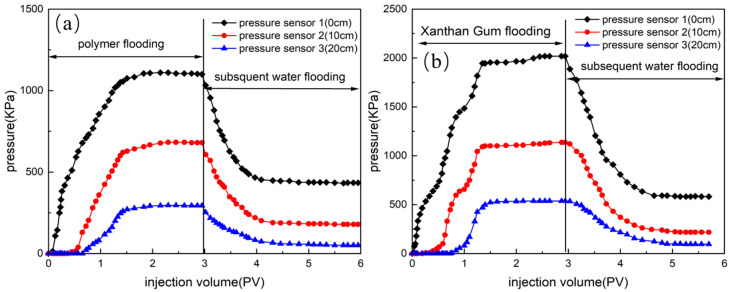
Variation in injection pressure with injection volume ((**a**) HPAM; (**b**) xanthan gum).

HPAM and xanthan gum with the same viscosity but different hydrodynamic radii were injected into the sand-packed model to investigate the effect of throat–polymer ratio on migration behavior. Figure 11 shows the change in injection pressure of HPAM and xanthan gum in the sand-packed model with injection volume, and Table 5 lists the corresponding resistance coefficients and residual resistance coefficients. Figure 11 and Table 5 demonstrate that the pressure of xanthan gum during the entire injection process, as well as its corresponding resistance coefficient and residual resistance coefficient, are greater than those of HPAM. This shows that xanthan gum with a small throat–polymer ratio retains more molecular clusters at the pore throat when it migrates in porous media, resulting in a higher resistance coefficient and residual resistance coefficient. In conclusion, the migration resistance of polymer system in porous media is directly related to the throat–polymer ratio (hydrodynamic radius). Due to its stronger retention and plugging characteristics, xanthan gum with a small throat–polymer ratio has a better ability to expand the swept volume and prevent scour than HPAM. Similarly, Aliabadian et al. [45] have also concluded that the migration of polymers in porous media is related to the ratio of the radius of polymer molecule gyration to the size of porous media. A critical ratio exists between polymer molecule size and pore size, and when this ratio exceeds a certain threshold, polymer retention must be taken into account.

In summary, the throat–polymer ratio has an important influence on the migration characteristics of polymer systems in porous media. With a decrease in the throat–polymer ratio (reduced permeability or increased hydrodynamic radius of the polymer), the retention and plugging ability of the polymer is enhanced, increasing the ability of the polymer system to expand the swept volume and resist erosion.

### 3.5. Understanding of Polymer System Transport in Reservoir Porous Media

**Figure 12:**
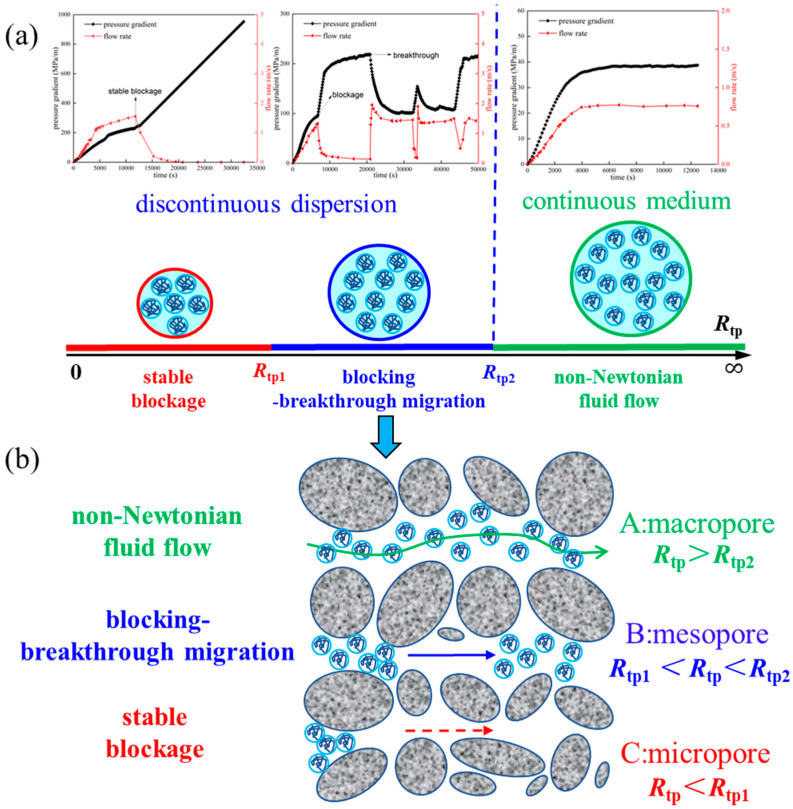
Migration patterns of polymer systems in porous media ((**a**) The migration of polymers in pore throat models; (**b**) The migration of polymers in porous medium).

For many years, petroleum engineers have measured viscosity with a rotational rheometer or a Brookfield viscometer in a millimeter-scale space, with viscosity representing the primary indicator for screening polymers for oil displacement. However, it must be emphasized that when the hydrodynamic radius of the polymer system used for oil displacement is similar to the radius of the reservoir pore throat (*R_tp_* < *R_tp_*_2_), the migration of the polymer system in the pore throat no longer exhibits the stable flow characteristics of a continuous medium. Rather, it is characterized by the migration of discontinuous dispersions (Figure 12a)—that is, temporary plugging-breakthrough migration and stable plugging of the polymer dispersion (Figure 12a). In other words, when the polymer’s size approaches that of the pore throat, the polymer system can no longer be considered to exhibit the flow of the polymer solution in the micron-scale porous medium of the reservoir; rather, during migration, the polymer clusters are carried by water as a dispersion through the narrow reservoir pore throats. Therefore, the migration of polymer systems in the narrow pores of a reservoir is fundamentally different (in the order of millimeters) from the flow in the measuring cylinder of a rheometer. The viscosity measured by a rheometer cannot accurately describe the dynamic behavior of the migration of real polymer systems in reservoir pores.

In summary, there are three situations in which a polymer system is injected into a reservoir porous medium (Figure 12b): Firstly, when the reservoir pores are macropores relative to the polymer system (*R_tp_* > *R_tp_*_2_), the macro rheology of the polymer system plays a dominant role in the migration characteristics of the polymer system. The macroscopic rheology of the polymer system can be used to predict its migration characteristics in the porous medium of a reservoir. Secondly, when the reservoir pores are mesopores relative to the polymer system (*R_tp_*_1_ < *R_tp_* < *R_tp_*_2_), the retained plugging-breakthrough migration effect of polymer clusters dispersed in the pore throat plays a leading role in the migration characteristics of the polymer system. This may be the main reason for the unusually high transport resistance of polymer systems in the reservoir. Thirdly, when the reservoir pores are micropores relative to the polymer system (*R_tp_* < *R_tp_*_1_), the stable blockage effect of polymer clusters dispersed in the pore throat plays a leading role in the migration characteristics of the polymer system. This will lead to a continuous increase in polymer injection pressure and damage the reservoir near the well. It is well known that the pore structure of reservoir porous media is diverse, and the three abovementioned migration states coexist in the process of injecting a polymer system into a deep reservoir. Therefore, both non-Newtonian flow and dispersion migration take place when a polymer system flows through a reservoir porous medium. In other words, polymer system migration resistance and improved sweep efficiency in porous media occur not only because of the viscosity of polymer, but also due to the blocking-breakthrough retention effect of polymer clusters. In particular, at the microscopic scale, the viscosity enhancement of polymers in porous media will significantly decrease [25]. Therefore, it is speculated that the blocking and retention effects of polymers in porous media may be more important reasons for the improved sweep efficiency of polymer flooding.

## 4. Conclusions

In this study, the effect of concentration on the viscosity and hydrodynamic radius of polymer systems was investigated using rheometry and dynamic light scattering. In addition, pore-throat models, homogeneous cores, and multi-measuring-point sand-packed models were constructed to investigate pore-scale migration patterns and the influence of the throat-to-polymer ratio on the transport properties of polymers in porous media. Finally, new insights into polymer system migration in reservoir porous media are presented. The main conclusions are as follows:

1. The higher the concentration of the polymer system, the denser its structure in water, and the higher its hydrodynamic radius and viscosity.
2. The throat–polymer ratio (*R_tp_*) is an important parameter influencing the migration behavior of polymer systems in pore throats. When *R_tp_* is sufficiently small, the migration pattern of the polymer system in the pore-throat model no longer exhibits the migration characteristics of polymer solution flow. Instead, it displays features of discontinuous-dispersion retention, plugging-breakthrough migration, and stable-plugging retention.
3. When the injection rate is increased, the polymer system exhibits the migration characteristics of discontinuous dispersion at a larger throat–polymer ratio.
4. Polymer system migration resistance and improved sweep efficiency in porous media are influenced by not only the viscosity of the polymer, but also the blocking-breakthrough retention effect of polymer clusters. As the throat–polymer ratio decreases, the retention and plugging ability of the polymer are enhanced, and the ability of the polymer system to expand the swept volume and resist erosion is improved.

It is worth noting that the findings of this study are solely based on experiments conducted with partially hydrolyzed polyacrylamide. While the current study advances our understanding of polymer transport in porous media, future work will focus on conducting a more comprehensive statistical survey incorporating a wider range of polymer systems under varied conditions.

## Figures and Tables

**Table 1:** Key parameters of the experiment.

Serial Number	Concentration (mg/L)	Hydrodynamic Radius (μm)	Injection Rate (mL/min)	Throat Radius (μm)	Throat–Polymer Ratio
1	50	0.396	0.02	25	63.13
2				20	50.50
3				15	37.88
4				12.5	31.57
5				10	25.25
6				7.5	18.94
7	100	0.531	0.02	25	47.08
8				20	37.66
9				15	28.25
10				12.5	23.54
11				10	18.83
12				7.5	14.12
13	50	0.396	0.04	25	63.13
14				20	50.50
15				15	37.88
16				12.5	31.57
17				10	25.25
18				7.5	18.94
19	100	0.531	0.04	25	47.08
20				20	37.66
21				15	28.25
22				12.5	23.54
23				10	18.83
24				7.5	14.12

**Table 2:** Core parameters.

Core Number	Permeability (μm^2^)	Porosity (%)	Diameter (mm)	Length (mm)	Average Throat Radius (μm)
C1	0.095	19.68	25.0	100.2	1.96
C2	0.155	18.70	24.9	98.7	2.58
C3	0.202	19.14	25.2	100.7	2.91
C4	0.243	20.13	25.1	100.4	3.11
C5	0.173	19.20	24.9	99.4	2.68
C6	0.267	19.60	25.0	100.3	3.30
C7	0.338	20.50	25.1	100.5	3.63
C8	0.390	21.80	25.0	99.7	3.78

**Table 3:** Parameters of sand-packed models.

Sand-Packed Model	Length (mm)	Diameter (mm)	Permeability, *k* (μm^2^)	Porosity, ∅ (%)	Average Throat Radius (μm)
S1	300	15	2.23	47.50	6.13
S2	300	15	2.19	46.10	6.16

**Table 4:** Resistance coefficients and residual resistance coefficients.

HPAM Concentration (mg/L)	Viscosity (mPa·s)	Hydrodynamic Radius (μm)	Permeability (μm^2^)	Resistance Coefficient	Residual Resistance Coefficient	*R_tp_*
50	8.59	0.396	0.095	blockage	blockage	4.96
			0.155	32.12	7.70	6.50
			0.202	25.85	5.85	7.33
			0.243	19.81	3.42	7.84
100	16.73	0.531	0.173	blockage	blockage	5.05
			0.267	55.18	13.30	6.21
			0.338	46.89	11.02	6.83
			0.390	37.24	7.29	7.13

**Table 5:** Resistance coefficient and residual resistance coefficient.

Chemical	Concentration (mg/L)	Viscosity (mPa·s)	Throat–Polymer Ratio	Distance (cm)	Resistance Coefficient	Residual Resistance Coefficient
xanthan gum	3000	92.41	4.07	0	360.54	103.94
				10	304.38	58.61
				20	288.39	51.59
HPAM	700	90.34	6.11	0	200.41	78.98
				10	185.61	49.00
				20	160.41	28.01

## Data Availability

The original contributions presented in this study are included in the article. Further inquiries can be directed to the corresponding author.

## Nomenclature

*R_tp_*	throat–polymer ratio (ratio of throat size to polymer hydrodynamic radius, non-dimensional)
*R_t_*	average throat radius, μm
*R_p_*	hydrodynamic radius of polymer system, μm
PV	pore volume, non-dimensional
*k*	core permeability, μm^2^
∅	core porosity, non-dimensional
$F_{R}$	resistance coefficient, non-dimensional
$F_{RR}$	residual resistance coefficient, non-dimensional
$∆p_{p}$	stable pressure of polymer displacement, KPa
$∆p_{w}$	the stable pressure of water displacement, KPa
$∆p_{sw}$	stable pressure of subsequent water displacement, KPa
